# Comparison of Pediatric Risk of Mortality-III, Phoenix Sepsis, and pediatric Sequential Organ Failure Assessment scores for predicting septic shock in Vietnamese children with sepsis

**DOI:** 10.1016/j.bjid.2026.104612

**Published:** 2026-01-20

**Authors:** Khai Quang Tran, Ngan Tuong Thien Pham, Tri Duc Nguyen, Quan Minh Pham

**Affiliations:** Department of Pediatrics, Faculty of Medicine, Can Tho University of Medicine and Pharmacy, Can Tho City, Vietnam

**Keywords:** Sepsis shock, PRISM III, Phoenix, Psofa, Vietnam

## Abstract

**Background:**

Early recognition of septic shock is crucial for improving outcomes in children with sepsis. This study aimed to compare the predictive performance of the Pediatric Risk of Mortality-III (PRISM-III), Phoenix Sepsis Score (PSS), and pediatric Sequential Organ Failure Assessment (pSOFA) scores for septic shock in Vietnamese children.

**Methods:**

A cross-sectional study was conducted on 86 children aged 2-months to 15-years with sepsis (including 23 with septic shock) admitted to a pediatric intensive care unit. Septic shock classification was performed independently and single ‒ blinded to score calculations to minimize assessment bias. The PSS and pSOFA were calculated using the worst parameters within the first 6-hours, and PRISM-III within the first 24 hours of admission. Discriminatory ability was assessed by the Area Under the Receiver Operating Characteristic Curve (AUROC). Multivariable logistic regression and calibration analyses were performed. Calibration results should be interpreted cautiously due to the small sample size.

**Results:**

The PSS showed the highest AUROC (0.867, 95 % CI: 0.777–0.931), followed by PRISM-III (0.826, 95 % CI: 0.729–0.899) and pSOFA (0.791, 95 % CI: 0.690–0.871); pairwise comparisons were not statistically significant. The PSS demonstrated the highest sensitivity (95.7 %) and negative predictive value (97.6 %), while PRISM-III had the highest specificity (90.5 %) and positive predictive value (70.0 %). In multivariable analysis, both PSS (Odds Ratio, OR = 2.78) and PRISM-III (OR = 1.23) were independent predictors of septic shock.

**Conclusions:**

The PSS and PRISM-III provide complementary value. A two-step approach using the sensitive PSS for initial screening and the specific PRISM III for confirmation may enhance early septic shock recognition in resource-limited settings.

## Introduction

Septic shock remains a life-threatening complication of pediatric sepsis, demanding timely recognition and intervention to mitigate its high mortality burden.^1^ The effective stratification of patients at greatest risk is therefore a critical priority in the management of critically ill children. Several scoring systems have been developed to quantify illness severity and organ dysfunction. The pediatric Sequential Organ Failure Assessment (pSOFA) was developed to grade organ dysfunction in children and has shown mortality prediction comparable to or better than prior scores in validation studies.^2^^,^^3^ Automated EHR-based pSOFA calculators have been developed and externally validated, facilitating scalable implementation.^4^ The Pediatric Risk of Mortality-III (PRISM-III) score, based on a comprehensive set of physiological and laboratory variables obtained within the first 24-hours of Pediatric Intensive Care Unit (PICU) admission, is a well-validated tool for predicting mortality risk in diverse settings.^5^^,^^6^ Recently, an international expert group proposed the Phoenix Sepsis Score (PSS) as a more sensitive diagnostic tool for pediatric septic shock than legacy SIRS-based criteria. Phoenix captures respiratory, cardiovascular, coagulation, and neurologic dysfunction; a threshold of Phoenix ≥ 2-points in suspected infection is closely associated with higher in-hospital mortality.^1^^,^^7^

While these scores are established for mortality prediction and sepsis definition, their comparative performance for the specific outcome of septic shock ‒ a crucial intermediary event ‒ is less clearly defined, especially in resource-limited contexts. In Vietnam, a concurrent, head-to-head evaluation of PRISM III, pSOFA, and PSS for the early prediction of pediatric septic shock is currently lacking. Such a comparison is essential to inform local clinical decision-making and resource allocation.

This study therefore aimed to fill this gap by assessing and comparing the predictive performance of the PRISM III, pSOFA, and PSS for septic shock in a cohort of children admitted to the PICU of Can Tho Children's Hospital, a key tertiary referral center in the Mekong Delta region of Vietnam.

## Material and methods

### Study design and setting

This cross-sectional diagnostic study was conducted at the PICU of Can Tho Children's Hospital, a tertiary referral center in the Mekong Delta of Vietnam, from May 2024 to May 2025.

### Study population and eligibility

A convenience sample of children aged 2-months to 15-years admitted to the PICU with a clinical diagnosis of sepsis was enrolled. Sepsis was defined according to the 2020 Surviving Sepsis Campaign guidelines, requiring suspected infection accompanied by cardiovascular dysfunction.^8^ Clinically, infection was indicated by fever (temperature > 38.5°C) or hypothermia (< 36°C), age-inappropriate tachycardia or tachypnea, or an identifiable infectious focus, along with signs of a systemic inflammatory response such as leukocytosis, leukopenia, or an increased proportion of band forms. Cardiovascular dysfunction was defined by the presence of at least one of the following criteria: persistent hypotension unresponsive to initial fluid resuscitation (≥ 40‒60 mL/kg in the first hour), the need for vasoactive support to maintain age-appropriate mean arterial pressure, or the presence of two or more clinical signs of inadequate perfusion (e.g., prolonged capillary refill, weak or absent peripheral pulses, a central-to-peripheral temperature gap > 3°C, elevated lactate, urine output < 0.5 mL/kg/h, or altered mental status) despite fluid and vasoactive therapies.

Patients were excluded if they had pre-existing chronic comorbidities, including viral-induced acquired immunodeficiency, thalassemia, nephrotic syndrome, cancer, complex congenital heart disease, or other immunodeficiencies. Further exclusion criteria included death before complete data could be captured, incomplete medical charts that prevented the calculation of PSS, pSOFA, or PRISM-III scores, or inter-hospital transfers with missing external medical records.

### Sample size calculation

This study was a single-gate, cross-sectional investigation designed to assess diagnostic test accuracy. A convenience sampling method was employed. The sample size was determined using Buderer’s formula for specificity, targeting a 95 % confidence level (Zα/2​ = 1.96) with an allowable error margin (w) of 4 %.^9^ Based on an expected prevalence (p) of septic shock of 0.17, which was proxied from mortality rates in similar pediatric populations, and an anticipated high Specificity (Sp) of 0.97 for modern prognostic scores, the minimum required sample size was calculated to be 84 participants.^10^^,^^11^^,^^12^ A total of 86 consecutively eligible patients were ultimately enrolled.

### Data collection and variables

Upon PICU admission, demographic data, clinical features, and laboratory results were collected. The primary infectious focus was determined from clinical, laboratory, and imaging findings. Septic shock classification was performed independently and single ‒ blinded to score calculations to minimize assessment bias. Prognostic scores were calculated as follows: The PSS was used to identify sepsis (score ≥2) and septic shock by evaluating four organ systems.^1^ The pSOFA score assessed six organ systems, utilizing the SpO_2_​/FiO_2_​ ratio when arterial blood gas data were unavailable.^2^ The PRISM III score was calculated using the most severe values of 17 physiological variables recorded within the first 24 hours of PICU admission to estimate mortality risk.^5^ For temporal alignment, PSS and pSOFA scores were determined using the earliest available data within the first six hours, while PRISM-III utilized the worst values over the first 24 hours.

Nutritional status was assessed using World Health Organization (WHO) z-scores: weight-for-length/height for children aged 0–59 months and BMI-for-age for those aged 5–15 years. For analysis, patients were categorized into a binary variable of either “Normal” or “Malnutrition/Overweight-Obesity” based on these *z*-scores.^13^^,^^14^ Tachypnea was defined using age-specific respiratory rate thresholds from WHO-Integrated Management of Childhood Illness (IMCI) guidelines for children under five years and American Heart Association Pediatric Advanced Life Support (PALS) guidelines for older children.^15^^,^^16^ The primary infectious focus was determined through a comprehensive review of clinical, laboratory, imaging, and microbiological data and was categorized accordingly. All data adjudication was independently performed by two investigators, with any discrepancies resolved through consensus.

### Statistical analysis

All data were cleaned, coded, and subsequently analyzed using Statistical Package for the Social Sciences (SPSS) version 22.0 (IBM Corp., Armonk, NY) and MedCalc version 22.017 (MedCalc Software Ltd., Ostend, Belgium).

Categorical variables were summarized as frequencies and percentages, with group comparisons conducted using the Chi-Squared (χ^2^) test or Fisher’s exact test where appropriate. The distribution of continuous variables was assessed for normality using the Shapiro-Wilk test, supplemented by visual inspection of histograms and Q-Q plots. Normally distributed data are presented as mean ± Standard Deviation (SD) and were compared using two-sided independent *t*-tests. Data that were not normally distributed are reported as median and Interquartile Range (IQR) and were compared using the Mann-Whitney *U* test.

To identify independent predictors, variables with a p-value less than 0.10 in univariable analysis, along with clinically prespecified variables, were entered into a multivariable logistic regression model. Results are presented as Odds Ratios (OR) with their corresponding 95 % Confidence Intervals (95 % CI). The discriminative ability of the models was evaluated using the Area Under the Receiver Operating Characteristic Curve (AUROC), with 95 % CIs. AUROCs were compared using the DeLong test. The optimal cut-off value for each model was determined using Youden’s index (J = sensitivity + specificity − 1). At these thresholds, we calculated sensitivity, specificity, Positive Predictive Value (PPV), Negative Predictive Value (NPV), and positive and negative likelihood ratios (LR+ and LR-).

**Fig. 1:**
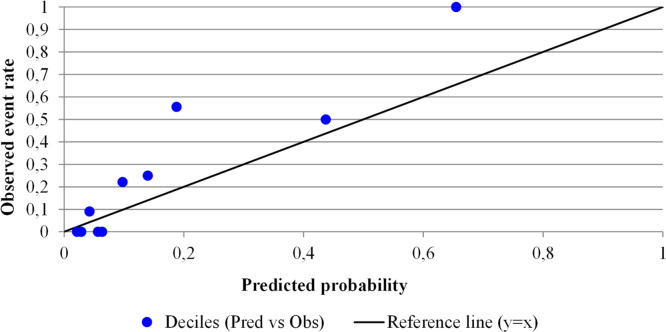
**Calibration plot comparing predicted vs observed shock risk by deciles.** Calibration plot comparing predicted versus observed septic shock risk by deciles for the exploratory multivariable model. Given the limited sample size, this calibration assessment is descriptive and should be interpreted cautiously.

Model calibration was assessed via the Hosmer-Lemeshow goodness-of-fit test, calibration-in-the-large (intercept), and the calibration slope, which are visually represented in Fig. 1. A two-sided p-value less than 0.05 was considered statistically significant for all analyses.

Given the limited number of septic shock events, multivariable logistic regression was performed for exploratory purposes only. Model estimates were interpreted cautiously due to the potential risk of overfitting and limited events-per-variable.

### Ethical approval statement

The study protocol was approved by the Institutional Review Board of Can Tho University of Medicine and Pharmacy (Approval n° 24.045.SV/PCT-HĐĐĐ, dated May 24, 2024). Informed consent was obtained from the parents or legal guardians of all participants.

## Result

### Study population and baseline characteristics

**Fig. 2:**
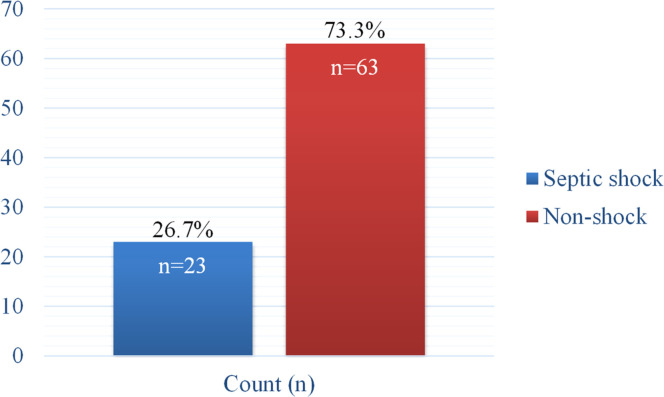
Classification of pediatric sepsis.

During the study period, a total of 86 children with sepsis were enrolled, of whom 23 (26.7 %) developed septic shock (Fig. 2).

The baseline characteristics of the cohort are presented in Table 1. Children older than 12-months had significantly higher odds of developing septic shock compared to those aged 2 to < 12-months (78.3 % vs. 21.7 %; OR = 3.07, 95 % CI: 1.01‒9.30, p = 0.041). No significant associations were found between septic shock and sex or nutritional status.

**Table 1:** Baseline characteristics of the study cohort.

Variable	Septic shock (n = 23)	Non-shock (n = 63)	Overall (n = 86)	OR (95 % CI)	p-value
**Age, n (%)**					
2 – < 12-months	5 (21.7 %)	29 (46.0 %)	34 (39.5 %)	3.071 (1.014‒9.297)	**0.041**
> 12-months	18 (78.3 %)	34 (54 %)	52 (60.5 %)		
**Sex, n (%)**					
Male	18 (78.3 %)	44 (69.8 %)	62 (72.1 %)	0.643 (0.208‒1.986)	0.441
Female	5 (21.7 %)	19 (30.2 %)	24 (27.9 %)		
**Nutritional status, n (%)**					
Malnutrition/ Overweight-obesity	9 (39.1 %)	24 (38.1 %)	38 (44.2 %)	0.396 (0.149‒1.054)	0.06
Normal	14 (60.9 %)	39 (61.9 %)	48 (55.8 %)		

### Clinical presentation and score distributions

**Fig. 3:**
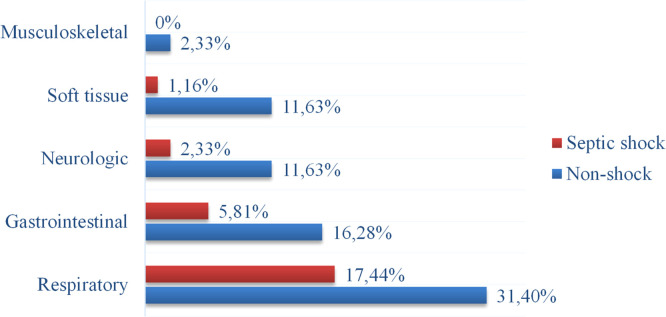
. Common infectious foci.

The distribution of primary infection foci differed between groups. Respiratory infections were the most common focus in both groups, but were more prevalent in the shock group (65.2 % vs. 42.9 %). Conversely, neurological and soft tissue foci were less common in the shock group (Fig. 3).

Fever at admission (≥ 38.5°C) was strongly associated with septic shock (91.3 % vs. 39.7 %; OR = 16.0, 95 % CI: 3.44‒74.13, p < 0.001), while the presence of tachypnea was not (78.3 % vs. 66.7 %; p = 0.41). As expected, the median scores for pSOFA (6 vs. 2, p < 0.001) and PSS (4 vs. 2, p < 0.001) were significantly higher in the shock group. The PRISM-III score was also higher in the shock group, but this difference did not reach statistical significance (9 vs. 7, p = 0.07) (Table 2).

**Table 2:** Clinical and laboratory characteristics.

Variable	Septic shock (n = 23)	Non-shock (n = 63)	p-value
Fever (≥ 38.5°C), n (%)	21 (91.3 %)	25 (39.7 %)	**<0.001**
Tachypnea, n (%)	18 (78.3 %)	42 (66.7 %)	0.410
pSOFA, median (IQR)	6 (3–8)	2 (0–4)	**<0.001**
PSS, median (IQR)	4 (3–6)	2 (2–3)	**<0.001**
PRISM III, median (IQR)	9 (6–14)	7 (5–9)	0.070

pSOFA, Pediatric Sequential Organ Failure Assessment; PSS, Phoenix Sepsis Score; PRISM-III, Pediatric Risk of Mortality-III.

### Predictive performance and diagnostic accuracy

In multivariable logistic regression analysis, both PSS (OR = 2.78, 95 % CI: 1.43–5.38, p = 0.002) and PRISM-III (OR = 1.23, 95 % CI: 1.02–1.48, p = 0.029) were independent predictors of septic shock, while pSOFA was not (p = 0.984) (Table 3).

**Table 3:** Multivariable logistic regression for predicting septic shock.

Variable	B	S.E.	Wald	OR (95 % CI)	p-value
PRISM-III	0.207	0.095	4.757	1.23 (1.02‒1.48)	**0.029**
PSS	1.022	0.337	9.183	2.78 (1.43‒5.38)	**0.002**
pSOFA	0.003	0.163	0.000	1.00 (0.73‒1.38)	0.984
Constant	-6.311	1.349	21.882	0.002	<0.001

pSOFA, Pediatric Sequential Organ Failure Assessment; PSS, Phoenix Sepsis Score; PRISM-III, Pediatric Risk of Mortality-III.

The diagnostic performance of the scores at their optimal cut-offs is detailed in Table 4. The PSS, at a cut-off of 2.5, showed exceptional sensitivity (95.7 %) and negative predictive value (97.6 %), making it an excellent rule-out tool. In contrast, PRISM-III, at a cut-off of 12.5, exhibited high specificity (90.5 %) and a strong positive likelihood ratio (LR+ 6.41), rendering it a useful rule-in tool. The pSOFA score showed balanced but moderate sensitivity (69.6 %) and specificity (81.0 %).

**Table 4:** Diagnostic performance at optimal cut-offs.

Variable	Cut-off	AUROC (95 % CI)	Sensitivity (%)	Specificity (%)	LR+ (95 % CI)	LR- (95 % CI)	PPV (%)	NPV (%)
pSOFA	4.5	0.791 (0.690‒0.871)	69.6	81.0	3.66 (2.05–6.50)	0.38 (0.20–0.71)	57.1	87.9
PSS	2.5	0.867 (0.777‒0.931)	95.7	63.5	2.62 (1.87–3.67)	0.07 (0.01–0.47)	48.9	97.6
PRISM-III	12.5	0.826 (0.729‒0.899)	60.9	90.5	6.41 (2.79–14.64)	0.43 (0.26–0.72)	70.0	86.4

pSOFA, Pediatric Sequential Organ Failure Assessment; PSS, Phoenix Sepsis Score; PRISM-III: Pediatric Risk of Mortality-III. AUROC and 95 % CIs by DeLong test (MedCalc v22.017). LR±CIs by the log (Simel) method.

Note: Performance metrics at the data-derived optimal cut-offs (determined by Youden's index) are presented for exploratory comparison only. These values should be interpreted as preliminary and are not proposed as validated clinical thresholds.

**Fig. 4:**
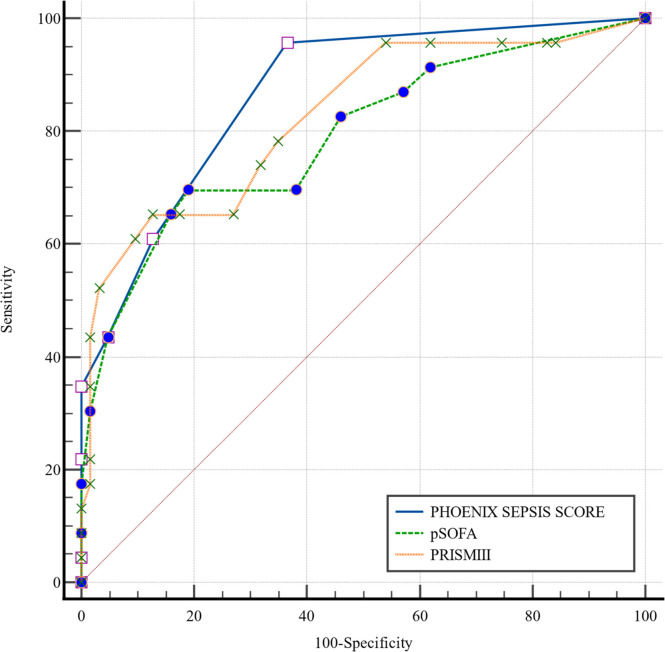
**ROC curves for pSOFA, PSS, and PRISM III.** Receiver Operating Characteristic (ROC) curves for the pediatric Sequential Organ Failure Assessment (pSOFA), Phoenix Sepsis Score (PSS), and Pediatric Risk of Mortality-III (PRISM-III) scores for the prediction of septic shock for exploratory purposes. The optimal cut-off values (determined by Youden's index) and associated performance metrics should be interpreted as preliminary and require validation in larger cohorts.

The PSS demonstrated the highest discriminative ability (AUROC = 0.867, 95 % CI: 0.777‒0.931), followed by PRISM-III (AUROC = 0.826, 95 % CI: 0.729‒0.899) and pSOFA (AUROC = 0.791, 95 % CI: 0.690‒0.871) (Fig. 4). However, pairwise comparisons revealed no statistically significant differences between the AUROCs (all p > 0.05, Table 5).

**Table 5:** DeLong pairwise comparisons of AUROCs.

Comparison	ΔAUC	95 % CI	z statistic	p-value
PSS vs. pSOFA	0.0763	-0.0220 → 0.174	1.522	0.128
PSS vs. PRISM-III	0.0418	-0.0820 → 0.165	0.661	0.508

pSOFA, Pediatric Sequential Organ Failure Assessment; PSS, Phoenix Sepsis Score; PRISM-III, Pediatric Risk of Mortality-III.

Note: Pairwise AUROC comparisons are presented for exploratory purposes.

### Model calibration and risk stratification

The multivariable model showed good calibration, with a non-significant Hosmer-Lemeshow test (χ^2^ = 10.64; p = 0.155) and a calibration slope of 0.814, indicating minimal systematic overfitting or underfitting (Fig. 1).

Stratification by the PSS cut-off of 2.5 effectively identified high-risk patients. The shock rate was 48.9 % in the high-score group (≥ 2.5) compared to only 2.4 % in the low-score group (< 2.5), corresponding to a markedly increased odds of shock (OR = 38.3, 95 % CI: 4.84‒302.79, p < 0.001) (Table 6).

**Table 6:** Risk stratification by phoenix sepsis score (cut-off 2.5).

Phoenix Sepsis Score	Shock	Non-shock	Total (n)	Shock risk (%)	OR (95 % CI)
	n (%)	n (%)			
< 2.5	1 (4.3 %)	40 (63.5 %)	41	2.4	Reference
≥ 2.5	22 (95.7 %)	23 (36.5 %)	45	48.9	38.261 (4.835–302.788)

## Discussion

This study provides the first direct comparison of the PSS, PRISM-III, and pSOFA for predicting septic shock in a Vietnamese pediatric cohort. The principal finding is that while all three scores demonstrate good discriminative capacity, their distinct performance characteristics suggest complementary, rather than interchangeable, clinical roles. The PSS emerges as an exceptional rule-out tool due to its high sensitivity, whereas PRISM-III serves as a potent rule-in instrument owing to its high specificity.

In our cohort, which had a septic shock prevalence of 26.7 %, the Phoenix Sepsis Score demonstrated the highest overall discrimination (AUROC = 0.867). At its optimal cut-off of ≥ 2.5, the PSS achieved an exceptional sensitivity of 95.7 % and a Negative Predictive Value (NPV) of 97.6 %. This profile, characterized by a very low negative likelihood ratio (LR- 0.07), establishes the PSS as an outstanding screening instrument. Its utility lies in reliably ruling out septic shock, thereby allowing clinicians to confidently de-escalate care for low-risk patients and focus resources on those requiring closer monitoring. This finding aligns perfectly with the intended purpose of the Phoenix criteria, which were designed to offer a more sensitive alternative to legacy SIRS-based definitions for early sepsis identification and risk stratification.^1^^,^^11^

Conversely, PRISM-III emerged as a powerful confirmatory tool. While its sensitivity was more modest (60.9 %), it delivered an excellent specificity of 90.5 % and a high positive likelihood ratio (LR+ 6.41). In a clinical context, a high PRISM III score substantially increases the post-test probability of septic shock, providing strong justification for escalating therapeutic interventions. This “rule-in” capability is consistent with PRISM-III's established role in assessing mortality risk and illness severity among critically ill children already admitted to the PICU.^5^

The pSOFA score occupied an intermediate position, with balanced sensitivity (69.6 %) and specificity (81.0 %). However, it failed to retain independent predictive value in the multivariable model when adjusted for PSS and PRISM-III. This suggests that the prognostic information captured by pSOFA may be largely redundant in the presence of the other two scores, reinforcing its utility as a general measure of organ dysfunction rather than a specialized tool for early septic shock prediction.^2^^,^^12^ These role distinctions are consistent with external evidence showing strong mortality-prediction performance of pSOFA and PRISM across settings.^17^, ^18^, ^19^ In parallel, emerging EHR-based models operationalize early shock risk in real time.^20^

While the DeLong test confirmed no statistically significant differences between the AUROCs of the three scores, their performance profiles are not interchangeable; rather, they are complementary.^21^ The results strongly support a pragmatic approach where the PSS, with its exceptional sensitivity and NPV (LR- 0.07), serves as the primary screening tool, aligning with its design for early and sensitive sepsis detection.^1^^,^^11^ Conversely, PRISM-III demonstrated higher specificity and positive predictive value (LR+ 6.41), suggesting stronger discriminative performance for identifying higher-risk patients within this cohort. This finding is consistent with its established role as a severity-of-illness score in critical care prognostication.^5^ These role distinctions are echoed by external validations showing Phoenix’s bedside utility.^22^^,^^23^ In parallel, robust mortality-prediction performance for pSOFA/PRISM across settings further supports a tiered, outcome-aligned strategy.^24^^,^^25^ In addition, real-time clinical decision support using EHR data has been shown to identify children at high risk for septic shock before onset.^26^ Pairwise AUROC comparisons showed no statistically significant differences. Therefore, the scores demonstrated broadly similar discriminative performance, with different sensitivity-specificity trade-offs.

These findings gain sharper clinical meaning when viewed alongside studies that explicitly model progression to septic shock rather than mortality endpoints. A time-evolving risk-score approach identified a distinct “pre-shock” state in pediatric sepsis and enabled early prediction hours before shock onset, supporting the idea that dynamic trajectories can warn of imminent deterioration.^27^ In the emergency setting, the diastolic/systolic blood pressure ratio (D/S) within the first 24-hours independently predicted progression to septic shock, with empirically derived cutoffs suitable for bedside triage.^28^ Prospective observational data from South Asia likewise found specific clinical and nutritional risk factors (e.g., severe acute malnutrition, culture positivity) that increase the odds of progressing to shock, highlighting context-sensitive risk features.^29^ Complementing these physiologic and clinical signals, a recent deep-learning model using electronic medical records estimated pediatric septic shock risk with high accuracy, illustrating how informatics can operationalize early warning in real time.^20^ Together, these reports reinforce our tiered interpretation of Phoenix ‒ using a lower threshold for early warning and a higher threshold for mortality enrichment ‒ while emphasizing the value of outcome-specific thresholds in practice.

### Limitations

The single-center design and modest sample size (n = 86), with only 23 septic shock events, limit statistical power and increase the risk of unstable estimates due to a low events-per-variable ratio. Accordingly, multivariable regression results should be interpreted with caution and considered exploratory and hypothesis-generating rather than confirmatory. Calibration analyses were also descriptive and should be interpreted cautiously. Cut-offs derived using Youden’s index are optimistic and should not be interpreted as clinically actionable thresholds without external validation.

Convenience sampling may introduce selection bias and limit the generalizability of our findings to the broader Vietnamese pediatric population. In addition, differences in score timing and clinical management may introduce incorporation bias. Specifically, time-varying therapies administered between early scoring (Phoenix Sepsis Score and pSOFA within the first 6-hours) and 24-hour PRISM III assessment may influence both shock classification and score components. Furthermore, several variables used to define septic shock overlap with components of Phoenix and pSOFA, which may artificially inflate discrimination metrics and limit the interpretability of comparative performance; this is one of the main limitations of the study. Future multicenter studies with larger samples, preregistered thresholds, and external validation (with planned subgroup analyses by age/infectious focus) are warranted. Similar single-center constraints have been highlighted in a Mekong Delta PICU cohort evaluating biomarker-based prognostication, reinforcing the need for broader validation.^30^

## Conclusion

In this Vietnamese pediatric cohort, exploratory findings suggest that the PSS and PRISM III may demonstrate complementary value for the early assessment of septic shock. The PSS, with its high sensitivity, demonstrates potentials as an initial screening tool to rule out shock. In contrast, PRISM-III, with its high specificity, appears valuable for confirming the diagnosis and supporting decisions to escalate care. These observations support the hypothesis of a two-step approach, utilizing the PSS within the first 6 hours followed by PRISM III within 24 hours. However, as this specific algorithm was not directly tested in the current study, it requires validation in larger multicenter trials prior to clinical implementation in resource-limited PICUs.

## Source(s) of support

None.

## Disclaimers

None.

## Data availability statement

The data that support the findings of this study are available from the corresponding author upon reasonable request.

## Conflicts of interest

The authors declare no conflicts of interest.
